# Studies on the Mechanism of Cu(II) Ion Sorption on Purolite S 940 and Purolite S 950

**DOI:** 10.3390/ma14112915

**Published:** 2021-05-28

**Authors:** Weronika Sofińska-Chmiel, Dorota Kołodyńska, Agnieszka Adamczuk, Aleksander Świetlicki, Marta Goliszek, Radosław Smagieł

**Affiliations:** 1 Analytical Laboratory, Institute of Chemical Sciences, Faculty of Chemistry, Maria Curie Skłodowska University, Maria Curie Skłodowska Sq. 2, 20-031 Lublin, Poland; goliszek@umcs.pl; 2 Department of Inorganic Chemistry, Institute of Chemical Sciences, Faculty of Chemistry, Maria Curie Skłodowska University, Maria Curie Skłodowska Sq. 2, 20-031 Lublin, Poland; dorota.kolodynska@mail.umcs.pl; 3 Institute of Agrophysics PAS, Doświadczalna 4 Str., 20-290 Lublin, Poland; a.adamczuk@ipan.lublin.pl; 4 Department of Materials Engineering, Mechanical Engineering Faculty, Lublin University of Technology, Nadbystrzycka 36 Str., 20-618 Lublin, Poland; aleksander.swietlicki@pollub.edu.pl; 5 Biolive Innovation, B. Dobrzańskiego 3 Str., 20-262 Lublin, Poland; radoslaw.smagiel@gmail.com

**Keywords:** heavy metals, sorption mechanism, ion exchangers, copper, Purolite S 940, Purolite S 950

## Abstract

The aim of the presented research was to investigate the mechanism of sorption of Cu(II) ions on the commercially available Purolite S 940 and Purolite S 950 chelating ion exchangers with the aminophosphonic functional groups. In order to understand better the sorption mechanism, the beads were cut with an ultramicrotome before and after the Cu(II) ion sorption process. The cut beads were examined by scanning electron microscopy (SEM) with an EDX detector. The performed linear profiles of the elemental composition allowed us to examine the depth with which the sorbed metal penetrates into. For further investigations concerning the mechanism of the sorption process, the Fourier transform infrared spectroscopy (FTIR) analysis using the attenuated total reflectance (ATR) technique and the X-ray photoelectron spectroscopy (XPS) methods have been used. The comparison of FTIR and XPS spectra before and after the sorption of Cu(II) ions showed that free electron pairs from nitrogen and oxygen in the aminophosphonic functional groups participate in the process of copper ion sorption. In addition, the microscopic studies suggested that the process of ion exchange between Na(I) ions and sorbed Cu(II) ions takes place on the Purolite S 940 and Purolite S 950. This study concerning the in-depth understanding the of Cu(II) sorption mechanism, using modern analytical tools and research methods could be very useful for its further modifications leading to the improvement of the process efficiency.

## 1. Introduction

Nowadays due to growing industrialization, society has to face various environmental problems. Among them, one of the greatest priorities is water pollution caused by heavy metal ions [1,2]. Metals of a density larger than 4.5 × 10^−3^ g/cm^3^ belong definition to the heavy metal group [2,3]. In chemical reactions, they tend to donate electrons forming simple cations. They show good thermal and electrical conductivity in the solid as well as liquid states, have a gloss, and are opaque. They have high melting and boiling points and are characterized by reducing properties [4,5]. Some of these heavy metals like copper are required by living organisms in a smaller quantity but at higher concentrations, its presence results in toxic effects [6]. They are found in raw sewage and they are not degraded in the sewage treatment being very harmful to living organisms causing mutagenic changes, damage to the central nervous system, and cancer [7,8,9,10]. Among them, copper which is present in fertilizers, tanning, and photovoltaic cells can cause allergies, cystic fibrosis, adreno-corticol hyperactivity, alopecia, arthritis, diabetes, hemorrhaging, and kidney disorders [11]. Copper is considered to process carcinogenic properties through the interaction of its ions with lipids finally resulting in DNA and tissue damage [12]. Therefore, one of the most important activities to protect human health and the natural environment is the improvement of water quality and sewage management [13,14,15,16].

Among different methods for wastewater treatment, ion exchange can be considered as one of prime importance as it is technologically simple and enables efficient removal of even traces of impurities from solutions [17,18,19].

The main advantage of using the ion exchange process for the electroplating treatment is that it has the greatest metal ion removal efficiency compared to the conventional methods such as reverse osmosis, electrolysis, or evaporation. At the same time, the ion exchange process requires the use of the smallest amount of energy for wastewater treatment compared to the above-mentioned methods. However, the disadvantage of this method is the need to use chemical reagents in the wastewater treatment process.

The use of natural sorbents would be a much more ecological solution. Yet, a much smaller efficiency of metal ion removal is obtained and is associated with some technical problems such as flow resistance through the ion exchange column.

The ion exchange process is very often used in many stages of technological processes in electroplating. They apply regeneration of chromium plating bath and pickling in phosphoric acid(V), regeneration of rinse water, regeneration of baths for passivation of zinc coatings, recovery of precious metals from rinse waters, final polishing of neutralized sewage. The selective ion exchangers with the aminophosphonic functional groups play a special role in the processes of galvanic wastewater treatment. They are used to remove residues that were not removed during treatment with conventional physicochemical methods.

In the wastewater treatment process, chelating ion exchangers are commonly used. A special feature of this type of resins is the ability of selective sorption of one metal ion in the presence of others. Selective sorption of metal ions is usually not possible using typical cation exchangers because the affinity for the metal ion present in the solution is only determined by the electrostatic interactions. The ability of selective sorption of transition metal ions allows the use of chelating ion exchangers in industrial wastewater treatment processes and recovery of valuable metals from ores and sludges [20,21,22].

As follows from the literature data, the chelating ion exchangers containing aminophosphonic functional groups are characterized better performance in the process of removing Cu(II) and Zn(II) ions from industrial waste, compared to other chelating resins [23]. They also have a weak affinity for Ca(II) and Mg(II) ions. An example of this type of materials is commercially available Purolite S 940 and Purolite S 950. There are numerous papers and research works concerning the sorption studies of copper ions [14,15,16], however, there is a lack of knowledge concerning the in-depth understanding of the mechanism of this process which can improve its efficiency greatly.

In the present study, the mechanism of Cu(II) ion sorption was examined based on the chosen ion exchangers using modern analytical techniques such as X-ray photoelectron spectroscopy (XPS), Fourier transform infrared spectroscopy (FTIR), and scanning electron microscopy (SEM). The presented results will allow us to learn more about the sorption mechanism using the chelating ion exchangers Purolite S 940 and Purolite S 950.

## 2. Materials and Methods

### 2.1. Material

**Figure 1:**
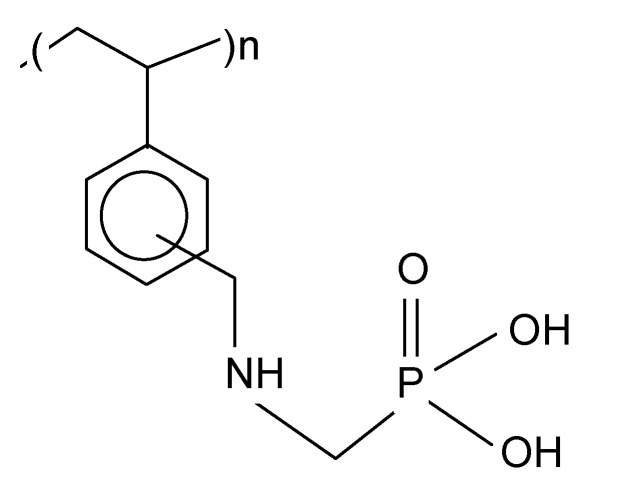
Chemical structure of Purolite S 940 and Purolite S 950.

Purolite S 940 and Purolite S 950 are macroporous chelating resins with the aminophosphonic functional groups. The matrix of the above-mentioned ion exchangers is polystyrene cross-linked with divinylbenzene. Their chemical structure is presented in Figure 1. The selected physicochemical parameters of Purolite S 940 and Purolite S 950 are presented in Table 1.

### 2.2. Methods

The research was carried out using the commercial ion exchangers Purolite S 940 and Purolite S 950 (Purolite Ltd., King of Prussia, PA, USA). The aim of the research was to select an appropriate commercial ion exchanger for polishing galvanic wastewater for electroplating plants. The research was carried out using the model solutions and real galvanic wastewater. This paper presents the results of the studies on the sorption of Cu(II) ions on Purolite S 940 and S 950 ion exchangers from the model 0.001 M CuCl_2_ solution.

The selection of the appropriate model concentration was preceded by the studies on the effect of the solution-ion exchanger phase contact time. Kinetic studies have also been carried out. These sorption tests were carried out using the following solutions on the Purolite S 940 and Purolite S 950 ion exchangers: 0.001 M CuCl_2_, 0.005 M CuCl_2_, 0.001 M CuSO_4_, 0.005 M CuSO_4_. In the process of removing Cu(II) ions, it should be stated that the chloride system is more favorable for the sorption process compared to the sulfate system with the initial concentration of Cu(II) ions amounting to 0.001 M. The tests proved that the equilibrium state for the solution with the concentration of 0.001 M is achieved in the case of sorption of the Cu(II) ions sorption on all tested ion exchangers after about 40 min. At 0.005 M concentration, equilibrium is reached in a shorter period of time. For example, for the Purolite S 950 ion exchanger, it is about 5 min. 0.001 M CuCl_2_ was selected as the model solution. The standard solution with a concentration of 0.001 M was prepared from the CuCl_2_∙2H_2_O salt (POCh S.A. Gliwice). Additionally, 63.54 g of salt was weighed and dissolved in 1 L of redistilled water. It was adjusted to pH = 4 with NaOH solution and HCl.

Kinetic parameters for the pseudo first-order and pseudo second-order equations as well as intraparticle diffusion were determined.

The linear relationship t/q_t_ = f(t) and the values of the determination coefficients R^2^ = 0.998 (close to unity), q_2_ = 8.17 mg/g, k_2_ = 0.076 g/mg min, h = 5.105 mg/g min as well as good agreement with the experimental data showed that the pseudo second-order kinetic model is fully suitable for the description of the Cu(II) sorption on Purolite S 940. For the IPD model the k_i_ values were equal to 0.259 [mg/g min^0.5^] and R^2^ = 0.829 for Purolite S 940. The same results were obtained for Cu(II) ions on the Purolite S 950 ion exchanger. The linear relationship t/q_t_ = f(t) and the values of the determination coefficients R^2^ = 0.998 (close to unity), q_2_ = 9.72 mg/g, k_2_ = 0.048 g/mg min, h = 4.537 mg/g min. For the IPD model the k_i_ value was equal to 0.259 [mg/g min^0.5^] and R^2^ = 0.829. Good agreement with the experimental data showed that the pseudo second-order kinetic model is fully suitable for the description of the sorption process.

The final pH was in the range of 4–5.5. The final concentration of the copper solution after sorption on Purolite S 940 ion exchanger was: 20.09 mg cm^−3^ and for Purolite S 950 ion exchanger was: 14.15 mg cm^−3^.

The process of Cu(II) ions sorption on Purolite S 940 and Purolite S 950 was conducted in 100 mL Erlenmeyer flasks shaken by means of a laboratory shaker type 357 (Elpin Plus) with a rotation rate of 180 rpm. 0.20 g of the resin was added to 20 mL of 0.001 M CuCl_2_ metal ion solution. After being shaken at 298 K for 2 h, the solution was separated from the resin. The resin was dried at room temperature.

#### 2.2.1. Microscopic Studies

In order to examine the chemical structure and explain the mechanism of Cu(II) ion sorption on Purolite S 940 and Purolite S 950, the beads were cut through by means of ultramicrotome EM UC7 (Leica) and examined using the optical SMZ 1500 stereoscopic microscope (Nikon). To examine the distribution of elements and the sorbed metal Cu(II) ions, the linear profiles of elemental compositions for the cut beads after the Cu(II) sorption were prepared. In this stage of the study, the scanning electron microscope Quanta 3D FEG with the EDS/EBSD (FEI) system was used.

#### 2.2.2. Fourier Transform Infrared Spectroscopy with Attenuated Total Reflection (FTIR-ATR)

In order to investigate the structure of ion exchangers before and after the Cu(II) sorption, the FTIR-ATR spectra of Purolite S 940 and Purolite S 950 using the FTIR Nicolet 8700 spectrometer (Thermo Scientific, Waltham, MA, USA) were recorded. They were performed by means of an ATR method with a diamond crystal in the range of wave numbers 4000–400 cm^−1^ and the spectral resolution of 4 cm^−1^. The spectra were recorded directly from the surface of the samples at room temperature.

#### 2.2.3. X-ray Photoelectron Spectroscopy (XPS)

In order to examine the chemical structure and explain the mechanism of Cu(II) ion sorption on Purolite S 940 and Purolite S 950, XPS tests were made using the Ultra High Vacuum multi-chamber analytical system (Prevac). The tests were carried out for Purolite S 940 and Purolite S 950 before and after the Cu(II) ion sorption. After being fixed on a molybdenum carrier, the samples were degassed at room temperature to the high constant vacuum of about ~5 × 10^−8^ mbar, in the UHV system sluice. After their introduction into the analytical chamber of the system, the appropriate analysis was performed by means of the XPS spectroscopy method. AlKα monochromatic radiation was used as a source of photoelectrons. Photoelectrons were stimulated by X-ray of a characteristic line AlKα of 1486.7 eV energy, generated by a VG Scienta SAX 100 lamp with an aluminum anode with a VG Scienta XM 780 monochromator. The pressure in the chamber during the measurements was 2 × 10^−8^ mbar. The X-ray tube operating parameters were as follows: U = 12 kV, Ie = 30 mA. Photoelectrons were recorded by the hemispherical analyzer Scienta R4000. The measurements were made based on the following basic parameters: operating mode—sweeping, pass energy—200 eV, measured range of the binding energy of photoelectrons 0–1200 eV, measuring step 0.5 eV, collection time in a single step 0.2 s, and the number of iterations 5. The parameters of the analyzer for the high-resolution spectra were: operating mode sweeping, pass energy 50 eV, measuring step 0.1 eV, and collection time in a single step 0.667 s.

## 3. Results and Discussion

### 3.1. Microscopic Studies

**Figure 2:**
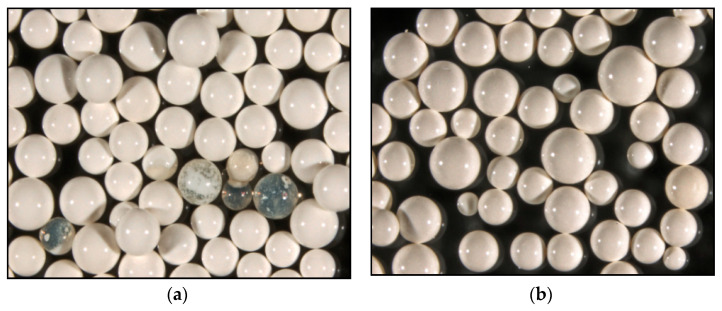
Photographs of (**a**) Purolite S 940, (**b**) Purolite S 950.

**Figure 3:**
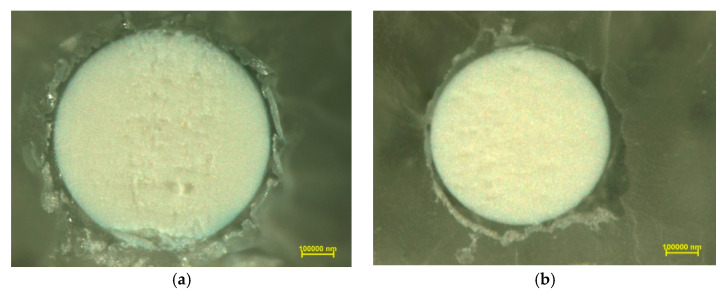
Photographs of cut (**a**) Purolite S 940, (**b**) Purolite S 950 after the Cu(II) ion sorption process.

**Figure 4:**
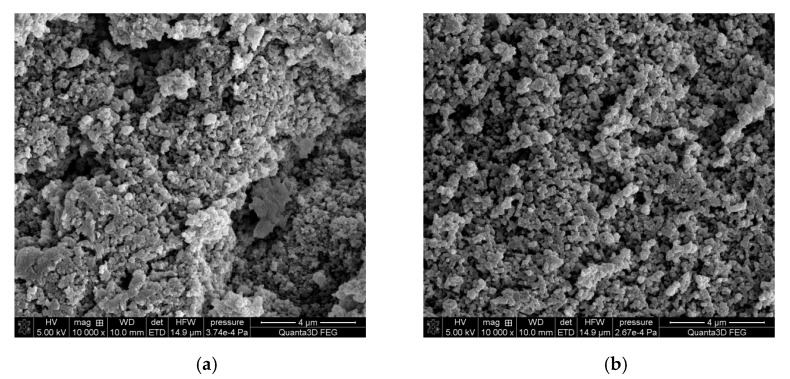
SEM images of cut (**a**) Purolite S 940, (**b**) Purolite S 950 after the Cu(II) ions sorption process.

Photographs of Purolite S 940 and Purolite S 950 were obtained using optical microscopy show a spherical shape and homogeneous structure of the examined ion exchangers (Figure 2 and Figure 3). The SEM microscopic photographs (Figure 4) allow us to observe the similarly developed porous structure of both. These tests are consistent with the BET-specific surface analysis using the Micrometitics ASAP 2420 apparatus (Table 1). The specific surface area for the Purolite S 940 ion exchange resin according to the BET method was 15.8 m^2^ g^−1^. whereas for the Purolite S 950 ion exchanger, it was 15.7 m^2^ g^−1^.

**Figure 5:**
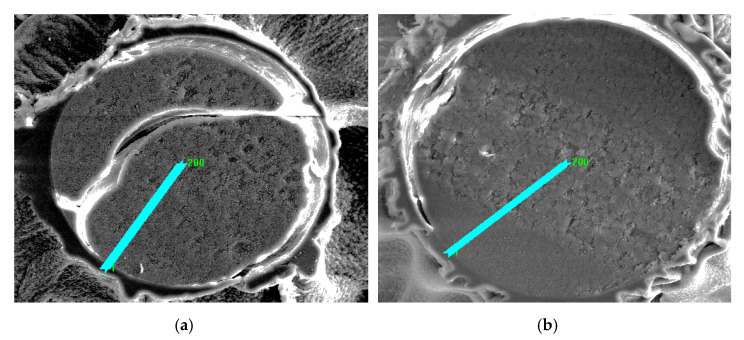
SEM images of lines for the analysis of the elemental composition of cut ion exchangers after the Cu (II) ion sorption process: (**a**) Purolite S 940, (**b**) Purolite S 950.

In order to investigate the distribution of elements, linear profiles of elemental composition were made. Figure 5 shows the microscopic photos of the cut ion exchanger beads on which the elemental composition analysis line was marked.

**Figure 6:**
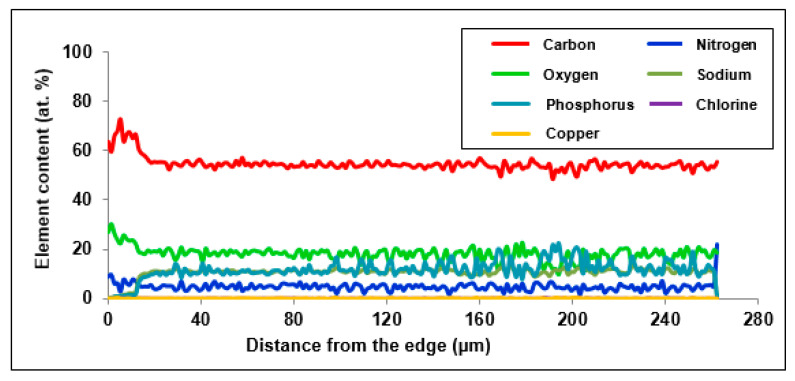
Distribution of the content of elements in the cut beads of Purolite S 940 after the Cu(II) ions sorption.

**Figure 7:**
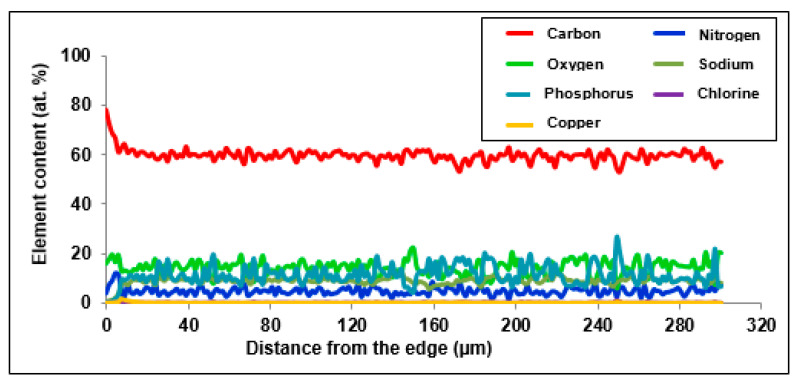
Distribution of the content of elements in the cut beads of Purolite S 950 after the Cu(II) ions sorption.

**Figure 8:**
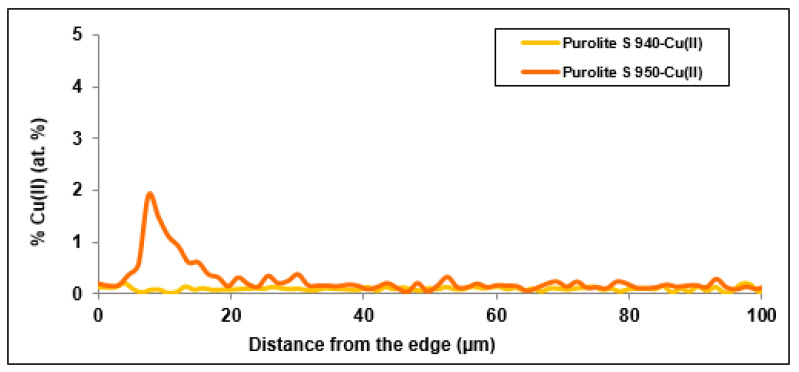
Distribution of copper ions in the cut beads of Purolite S 940 and Purolite S 950.

Figure 6 and Figure 7 present the distribution of elements present in the examined ion exchangers. In Figure 8, the comparison with particular emphasis on Cu(II) ions is presented.

The study of the linear profile of the elemental composition of Purolite S 940 allowed us to determine the distribution of copper ions along the entire length of the profile. As follows from the research, it can be concluded that the concentration of copper ions is evenly distributed over the entire length of the profile, with a slight increase in concentration on the surface of the tested grain. The thickness of the layer with the increased concentration is approx. 5 µm. A reduced sodium content was also observed on the grain surface in a layer with a thickness of about 12 µm (Figure 6). This may indicate the ion exchange process that takes place between Na(I) and Cu(II) ions. The distribution of the remaining elements is uniform along the entire length of the profile with a slight increase in the concentration of carbon and oxygen on the ion exchanger surface.

The study of the linear profile of the elemental composition of Purolite S 950 showed an increased content of absorbed copper ions on the grain surface. The thickness of the layer with the increased content of copper ions was approx. 16 µm. The maximum concentration in this layer was 1.9 at.% at a distance of approx. 9 µm from the edge of the ion exchanger. In the near-surface layer, where an increased concentration of copper ions was observed, the concentration of sodium ions decreased evidently. The thickness of the layer with the reduced content of sodium ions was approx. 10 µm (Figure 7). This phenomenon may suggest that the ion exchange process takes place between Na(I) and Cu(II) ions. The distribution of the remaining elements is practically uniform as is the case with Purolite S 940.

According to the literature data, the penetration depth of the sorbed Cu (II) ions on the Dowex M4195 chelating ion exchanger is approx. 40 µm [24]. In the case of the Purolite S 940 ion exchanger, the highest concentration of sorbed Cu (II) ions was observed at a distance of about 5 µm from the edge of the ion exchanger. In the case of the Purolite S 950 ion exchanger, the highest sorption of Cu (II) ions was observed for 16 µm from the edge of the ion exchanger. This was definitely a smaller penetration into the ion exchanger than in the case of Dowex M 4195. Smaller penetration into the ion exchanger grains was also observed compared with the galvanic wastewater sorption [25].

### 3.2. ATR-FTIR Analysis

**Figure 9:**
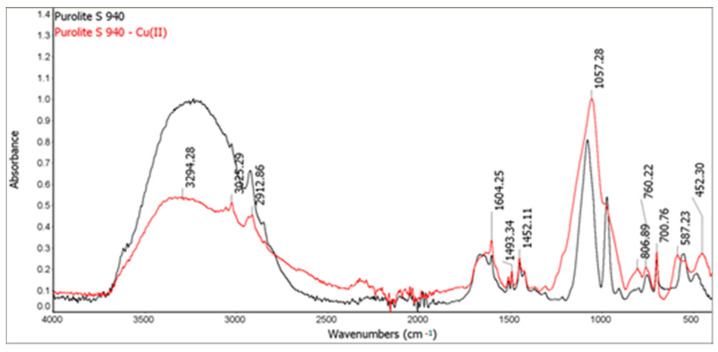
FTIR-ATR spectra of Purolite S 940 before and after the Cu(II) ion sorption process.

**Figure 10:**
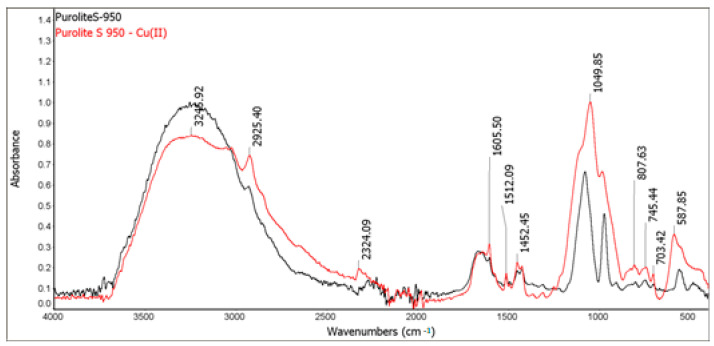
FTIR-ATR spectra of Purolite S 950 before and after the Cu (II) ion sorption process.

The FTIR spectra of the ion exchangers are presented in Figure 9 and Figure 10. Before the sorption process, a band of great intensity in the 3600–3200 cm^−1^ range was observed on the FTIR-ATR spectra of Purolite S 940 and Purolite S 950. This is related to the vibrations of the O-H and N-H groups. The bands in the range of 3000–2850 cm^−1^ correspond to the symmetric and asymmetric vibrations of aliphatic CH_2_ groups. Skeletal vibrations were also observed in the range of 1600–1585 cm^−1^ and 1500–1400 cm^−1^. The presence of bands related to the P=O stretching vibrations is observed in the range of 1350–1150 cm^−1^ and the P-OH group in the range of 1100–900 cm^−1^ [26]. The FTIR-ATR spectra also show the bands in the range 1250–1020 cm^−1^ derived from the C-N stretching vibrations of aliphatic amines. The bands in the range 730–675 cm^−1^, corresponding to the out-of-plane deformation vibrations of the C-H groups were also observed [27,28,29].

The studies of Purolite S 940 and Purolite S 950 after the Cu(II) ion sorption process showed significant band shifts in the range of 1250–900 cm^−1^. In this area, there are bands characteristic of the stretching vibrations of the P=O (1350–1150 cm^−1^) and P-OH (1100–900 cm^−1^) groups. Changes in the bands originating from the stretching vibrations of C-N groups present in aliphatic amines were also observed. These changes suggest the formation of a bond within the aminophosphonic functional group and Cu(II) ions.

### 3.3. XPS Analysis

The XPS analysis was performed in wide and narrow ranges of bond energies for Purolite S 940 before the Cu(II) ion sorption process and the results are presented in Table 2 and Table 3, respectively.

According to the presented results, 79.2% of the carbon present on the surface is in the form of C=C, C-C, and C-H binding. The other bonds are C-N. Of the total number of oxygen atoms, 62% are present in the form of -HPO_3_Na combinations derived from the aminophosphonic functional group. The XPS tests showed the presence of three forms of nitrogen: 58.0% of nitrogen atoms are in the form of an aliphatic amine, 19.2% comes from the N-H binding of intramolecular or extra-molecular hydrogen bonds, and 22.8% of nitrogen atoms are in the form of quaternary nitrogen [30]. It is also possible to form a hydrogen bond inside the aminophosphonic group between the hydrogen of the N-H group and the adjacent oxygen or between these elements in adjacent aminophosphonic groups (Table 3) [31,32,33,34,35,36,37,38,39]. The percentages of individual elements are given in atomic %.

The XPS analyses were also performed in a narrow and wide range of binding energies for Purolite S 950 before the Cu(II) ion sorption process. The results are presented in Table 4 and Table 5.

**Figure 11:**
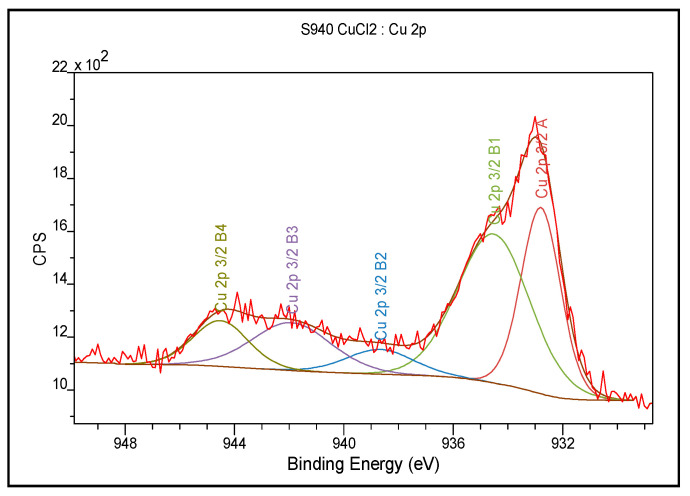
The XPS spectrum obtained in the narrow range of binding energy for Purolite S 940 after the Cu(II) ion sorption process.

**Figure 12:**
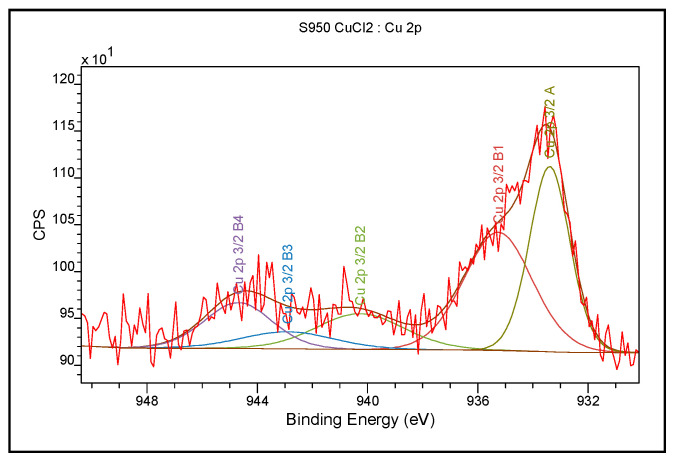
The XPS spectrum obtained in a narrow range of the binding energy for Purolite S 950 after the Cu(II) ion sorption process.

In the case of Purolite S 950, 81.4% of carbon present on the surface is in the form of C=C, C-C, C-H binding, while 18.6% of carbon is in the form of CN binding. Out of the total number of oxygen atoms, 69.9% is in the form of -HPO_3_Na binding derived from the functional aminophosphonic group, the other forms occur as the PO3^−^ and O-H bonds. As for nitrogen 67.7% is in the form of C-N bonds, 17.2% in the form of N-H bonds forming intramolecular or extra-molecular hydrogen bonds, and 15.1% of nitrogen atoms are in the form of quaternary nitrogen. Alike Purolite S 940, the formation of hydrogen bonds is not excluded [33,34,35,36,37,38,39]. In order to investigate changes in the chemical bonds of functional groups as a result of the sorption process, XPS tests were also performed for Purolite S 940 and Purolite S 950 after the Cu(II) ion sorption process. The test results for Purolite S 940 are presented in Table 6 and Table 7 and in Figure 11. For Purolite S 950, the results are presented in Table 8 and Table 9 and Figure 12.

For Purolite S 940 and Purolite S 950 after the Cu(II) sorption process, the bands of nitrogen atoms in the form of an aliphatic amine were shifted towards higher binding energies by 1 eV, i.e., from 399.2 eV to 400.2 eV for Purolite S 940 and 0.6 eV, i.e., from 399.1 to 399.7 for Purolite S 950. For the above-mentioned ion exchangers, there was also a shift of the band originating from the N-H binding forming on intramolecular or extra-molecular hydrogen bond from 400.3 eV to 402 eV and from 400.1 eV to 400.7 eV. There was also found the presence of three forms of copper: Cu(0), Cu(I), and Cu(II). After the sorption of Cu (II) ions, the XPS tests also showed a decrease in the sodium content on the surface of the Purolite S 940 ion exchanger. The sodium content was below the detection limit of the method, i.e., 0.1%. In the case of the Purolite S 950 ion exchanger, there was an observed decrease from 8.6 atomic % to 6.3 atomic % of the sodium content.

**Figure 13:**
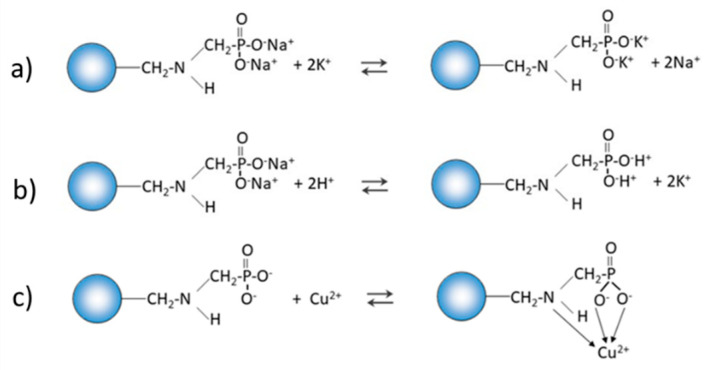
Mechanism of electrostatic interactions in ion exchangers with an aminophosphonic functional group.

Chelating ion exchangers, depending on the pH of the analyte sample, may undergo electrostatic interactions with metal ions. As an example, the reactions of the chelating ion exchanger with aminophosphonic functional groups are given in Figure 13.

**Figure 14:**
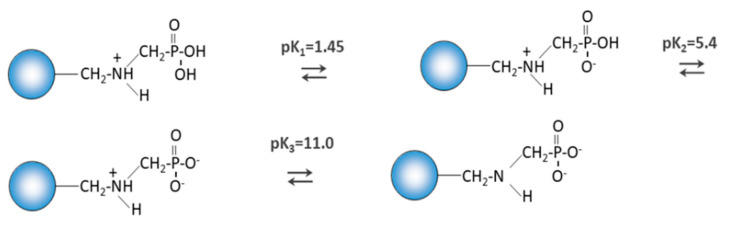
Forms of ion exchangers depending on the pH of the solution.

**Figure 15:**
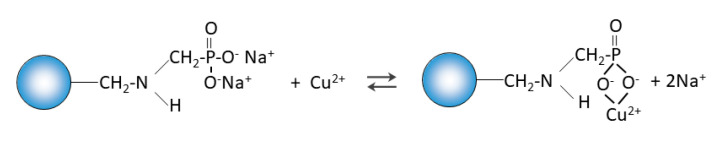
Forms of ion exchangers in acidic solution.

**Figure 16:**
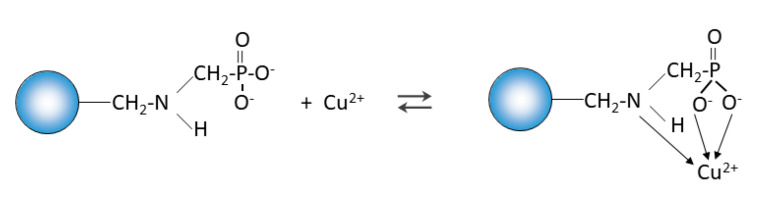
Proposed sorption mechanism.

**Figure 17:**
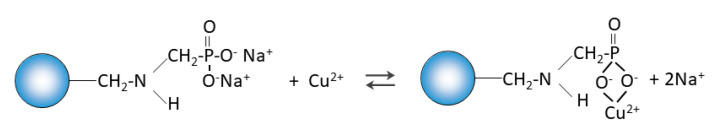
Possibility of ion exchange between Na(I) and Cu(II).

Chelating ion exchangers can therefore behave as typical ion exchangers (a), they can undergo protonation reactions (b) or the reaction of complexing metal ions (c) can take place in the sorption process. In addition, it should be noted that, depending on the pH, the aminophosphonic group can exist in the following forms (Figure 14):

In the case of acidic solutions, as a result of the protonation of the nitrogen atom of the aminophosphonic group in the process of sorption of metal ions, connections with the following structure are formed (Figure 15):

In the case of the conducted research, the participation of oxygen atoms in the sorption process of Cu (II) ions was observed as in Figure 15. Moreover, the XPS tests also showed the participation of nitrogen atoms in the formation of the bond with sorbed copper atoms, which made it possible to propose the following sorption process mechanism for Purolite S 940 and Purolite S 950 (Figure 16):

The microscopic studies showed the possibility of ion exchange between Na(I) and Cu(II) (Figure 17).

## 4. Conclusions

The studies with spectroscopic methods allowed for a comprehensive evaluation of the processes taking place during the sorption of Cu(II) ions on Purolite S 940 and Purolite S 950. The research allowed us to suggest the mechanism of Cu(II) sorption on the Purolite S 940 and Purolite S 950 chelating ion exchangers. The research confirmed that the sorption process takes place mainly on the surface of the examined ion exchangers.

The microscopic studies showed a different course of the Cu(II) sorption process on these ion exchangers. In the case of Purolite S 940, a uniform distribution of Cu(II) ion concentration was observed throughout the grain volume. In the case of Purolite S 950, the increased sorption of Cu(II) ions on the ion exchanger surface were demonstrated. The microscopic studies also showed the possibility of the ion exchange process between the Na(I) ions and the sorbed Cu(II) ions on Purolite S 940 and Purolite S 950.

The FTIR and XPS analyses exhibited some changes in the spectra of Purolite S 940 and Purolite S 950 before and after the process of Cu(II) ions sorption. These changes indicate the participation of nitrogen and oxygen present in the aminophosphonic functional groups in the sorption process. The research proved that in the process of sorption of Cu(II) ions on the chelating ion exchangers Purolite S 940 and Purolite S 950, both the ion exchange process and the formation of a coordination bond with the participation of free electron pairs of nitrogen and oxygen in the aminophosphonic functional groups take place.

The microscopic tests confirmed the kinetic test results in which the better efficiency of the Cu (II) ion sorption process was obtained for the Purolite S 950 ion exchanger.

The linear relationship t/q_t_ = f (t) and the values of the determination coefficients (R^2^) close to unity as well as good agreement with the experimental data showed that the pseudo second-order kinetic model is fully suitable for the description of the sorption process. The studies of the sorption of Cu(II) ions from the 0.001 CuCl_2_ solutions showed better process efficiency on Purolite S 950 ion exchanger (q_t_ = 9.88 mg g^−1^) compared to Purolite S 940 ionite (q_t_ = 8.66 mg g^−1^).

The research allows us to state that spectroscopic methods are effective in the sorption processes studies.

## Figures and Tables

**Table 1:** Selected physicochemical parameters of Purolite S 940 and Purolite S 950.

Ion Exchanger	Purolite S 940	Purolite S950
Manufacturer	Purolite International Ltd.	Purolite International Ltd.
Matrix	styrene-divinylbenzene copolymer	styrene-divinylbenzene copolymer
Structure	macroporous	macroporous
Functional groups	aminophosphonic	aminophosphonic
Physical form	white spherical grains	white spherical grains
Ionic form	Na^+^	Na^+^
Maximum working temperature	163.15 K	163.15 K
Specific gravity	1.11	1.13
Grain size	0.425–0.850 mm	0.3–1.2 mm
pH range	H^+^ form: 2–6 Na^+^ form: 6–11	H^+^ form: 2–6 Na^+^ form: 6–11
Specific surface area	15.8 m^2^/g	15.7 m^2^/g

**Table 2:** The elemental composition of Purolite S 940 determined by the XPS method.

Name	Position (eV)	FWHM (eV)	Raw Area	% Atom Concentration	% Mass Concentration
C 1s	284.7	2.26	45,983.8	57.0	42.8
O 1s	531.1	2.80	46,629.1	19.7	19.7
N 1s	398.7	2.31	4747.1	3.3	2.9
Na 1s	1071.7	2.39	65,380.1	9.5	13.7
P 2p	132.2	2.35	7246.6	7.6	14.6
Cl 2p	198.2	2.45	5302.6	2.9	6.4

FWHM–full width at half maximum.

**Table 3:** The XPS analysis results obtained in the narrow range of binding energies for Purolite S 940.

Name	Position [eV]	FWHM [eV]	Raw Area	% Atom Concentration	Phase
O 1s	530.8	1.58	3321.4	62.0	-HPO_3_Na
O 1s	532.3	1.50	1593.9	29.7	PO_3_^−^
O 1s	533.5	1.40	444.2	8.3	O-H
Na KLL	535.8	2.30	1308.2	-	-
C 1s	284.7	1.24	3598	79.2	C=C, C-C, C-H
C 1s	285.9	1.40	944.9	20.8	C-N
N 1s	399.2	1.21	289.1	58.0	C-N from an aliphatic amine
N 1s	400.3	1.27	87.5	19.2	N-H
N 1s	402.0	1.09	92.4	22.8	N quaternary
P 2p 3/2	132.3	1.58	513.1	100.0	-HPO_3_Na
P 2p 1/2	133.2	1.70	256.5	-	
Cl 2p 3/2	198.2	1.43	345.5	100.0	NaCl
Cl 2p 1/2	199.8	1.41	172.7	-	

**Table 4:** The elemental composition of Purolite S 950 determined by the XPS method.

Name	Position [eV]	FWHM [eV]	Raw Area	% Atom Concentration	% Mass Concentration
C 1s	284.7	2.26	51,190	56.8	54.9
Na 1s	1071.2	2.39	65,739.4	8.6	15.9
N 1s	398.7	2.34	7124.4	4.4	5.0
P 2p	132.7	2.58	10,396	9.7	24.2
O 1s	530.6	2.55	54,043.2	20.5	26.4

**Table 5:** The XPS analysis results were obtained in the narrow range of binding energies for Purolite S 950.

Name	Position (eV)	FWHM (eV)	Raw Area	% Atom Concentration	Phase
O 1s	530.8	1.47	3937.8	69.9	-HPO_3_Na
O 1s	532.2	1.45	1223	21.7	PO_3_^−^
O 1s	533.3	1.45	472.3	8.4	O-H
C 1s	284.7	1.32	3755	81.4	C=C, C-C, C-H
C 1s	285.8	1.4	857.4	18.6	C-N
N 1s	399.1	1.17	380.2	67.7	C-N from an aliphatic amine
N 1s	400.1	1.30	98.5	17.2	N-H
N 1s	401.8	1.30	90.1	15.1	N quaternary
Na 1s	1071.7	1.77	7112.4	100	-HPO_3_Na
P 2p 3/2	132.3	1.53	678.5	100	PO_4_^3−^
P 2p 1/2	133.3	1.70	339.2	-	-

**Table 6:** The elemental composition determined for Purolite S 940 after the Cu(II) ion sorption process.

Name	Position [eV]	FWHM [eV]	Raw Area	% Atom Concentration	% Mass Concentration
C 1s	284.7	2.31	55,326.2	64.2	47.6
O 1s	531.2	2.65	49,409	19.6	19.3
Cu 2p	932.7	3.83	63,978.2	2.9	11.5
P 2p	132.7	2.66	8811.87	8.6	16.4
N 1s	400.7	3.40	5819.9	3.8	3.2
Cl 2p	198.7	1.90	1779.63	0.9	2.0

**Table 7:** The XPS analysis results obtained in the narrow range of binding energies for Purolite S 940 after the Cu(II) ions sorption.

Name	Position (eV)	FWHM (eV)	Raw Area	% Atom Concentration	Phase
O 1s	531.2	1.50	2725.7	56.7	-HPO_3_Na
O 1s	532.1	1.42	1242.7	25.9	PO_3_^−^
O 1s	533.1	1.50	837.5	17.4	O-H
C 1s	284.7	1.41	4240.3	75.9	C=C, C-C, C-H
C 1s	285.9	1.38	900.9	16.1	C-N
C 1s	286.9	1.40	197.1	3.5	C-N
C 1s	291.5	2.30	249.9	4.5	π→π*
N 1s	400.2	1.60	148.7	45.0	C-N from an aliphatic amine
N 1s	402.0	1.58	139.4	38.5	N-H
N 1s	403.4	1.60	62.9	16.5	N quaternary
P 2p 3/2	132.9	1.65	576.6	100.0	PO_4_^3−^
P 2p 1/2	133.8	1.67	288.3	-	
Cl 2p 3/2	198.6	1.60	108.2	83.1	CuCl_2_
Cl 2p 1/2	200.2	1.42	54.1	-	
Cl 2p 3/2	201.6	1.29	22	16.9	
Cl 2p 1/2	203.2	1.60	11	-	
Cu 2p 3/2	932.7	1.30	954.4	22.0	Cu(0), Cu(I)
Cu 2p 3/2	934.3	3.00	2181.2	50.3	Cu(II)
Cu 2p 3/2	939	3.00	218.1	5.0	
Cu 2p 3/2	941.8	3.50	654.4	15.2	
Cu 2p 3/2	944	2.00	327.2	7.5	

**Table 8:** The Elemental composition determined for the Purolite S 950 after the Cu(II) ion sorption process.

Name	Position (eV)	FWHM (eV)	Raw Area	% Atom Concentration	% Mass Concentration
C 1s	284.7	2.36	25,887	57.6	43.3
Na 1s	1071.7	2.36	24,040.9	6.3	9.1
P 2p	132.7	2.33	5753.8	10.8	21.0
N 1s	399.2	3.94	3699.1	4.6	4.3
Cu 2p	933.7	2.96	6133.3	0.5	2.0
O 1s	531.2	2.61	26,609.4	20.2	20.3

**Table 9:** The XPS analysis results obtained in the narrow range of binding energy for Purolite S 950 after sorption of Cu(II) ions.

Name	Position (eV)	FWHM (eV)	Raw Area	% Atom Concentration	Phase
O 1s	530.7	1.42	1558.4	60.1	-HPO_3_Na
O 1s	531.7	1.60	690.2	26.6	(PO_3_)^−^
O 1s	532.9	1.54	343.9	13.3	O-H
C 1s	284.7	1.30	2032.6	81.0	C=C, C-C, C-H
C 1s	285.9	1.30	392.5	15.6	C-N
C 1s	291	2.01	83.6	3.3	π→π*
N 1s	399.3	1.32	212.0	61.5	C-N from an aliphatic amine
N 1s	400.7	1.38	43.8	12.8	N-H
N 1s	401.9	1.38	89.60	25.6	N quaternary
P 2p 3/2	132.5	1.59	348.6	100.0	PO_4_^3−^
P 2p 1/2	133.4	1.60	174.3		
Na 1s	1071.7	1.63	2623.2	100.0	NaCl
Cu 2p 3/2	933.2	1.75	362.2	36.0	Cu(0), Cu(I)
Cu 2p 3/2	935.2	2.82	399.9	39.7	Cu(II)
Cu 2p 3/2	940	2.82	48	4.7	
Cu 2p 3/2	942.7	3.67	160	15.2	
Cu 2p 3/2	944.6	1.69	44	4.4	

## Data Availability

The data presented in this study are available on request from the corresponding author.

## References

[1] Zhang M., Yin Q., Ji X., Wang F., Gao X., Zhao M. (2020). High and fast adsorption of Cd(II) and Pb(II) ions from aqueous solutions by a waste biomass based hydrogel. Sci. Rep..

[2] Briffa J., Sinagra E., Blundell R. (2020). Heavy metal pollution in the environment and their toxicological effects on humans. Heliyon.

[3] Turdean G.L. (2011). Design and development of biosensors for the detection of heavy metal toxicity. Int. J. Electrochem..

[4] Malik L.A., Bashir A., Qureashi A., Pandith A.H. (2019). Detection and removal of heavy metal ions: A review. Environ. Chem. Lett..

[5] Bashir A., Ahad S., Pandith A.H. (2016). Soft template assisted synthesis of zirconium resorcinol phosphate nanocomposite material for the uptake of heavy-metal ions. Ind. Eng. Chem. Res..

[6] Valko M., Morris H., Cronin M.T.D. (2005). Metals, toxicity and oxidative stress. Curr. Med. Chem..

[7] Ali H., Khan E. (2018). Bioaccumulation of non-essential hazardous heavy metals and metalloids in freshwater fish. Risk to human health. Environ. Chem. Lett..

[8] Dubey S., Shri M., Gupta A., Rani V., Chakrabarty D. (2018). Toxicity and detoxifcation of heavy metals during plant growth and metabolism. Environ. Chem. Lett..

[9] Zhu J., Fu Q., Qiu G., Liu Y., Hu H., Huang Q., Violante A. (2019). Influence of low molecular weight anionic ligands on the sorption of heavy metals by soil constituents: A review. Environ. Chem. Lett..

[10] Lair G.J., Gerzabek M.H., Haberhauer G. (2007). Sorption of heavy metals on organic and inorganic soil constituents. Environ. Chem. Lett..

[11] Selvaraj S., Krishnaswamy S., Devashya V., Sethuraman S., Krishnan U.M. (2011). Investigations on membrane perturbation by chrysin and its copper complex using self-assembled lipid bilayers. Langmuir.

[12] Mar A., Fa M., Salazar J., Mar R., Aracena P. (2005). Possible mechanisms underlying copper-induced damage in biological membranes leading to cellular toxicity. Chem. Biol. Interact..

[13] Gumpu M.B., Sethuraman S., Krishnan U.M., Rayappan J.B.B. (2015). A review on detection of heavy metal ions in water-An electrochemical approach. Sens. Actuator B Chem..

[14] Ho Y.S., Wase D.A.J., Forster C.F. (1994). The Adsorption of divalent copper ions from aqueous solution by *Sphagnum moss* peat. Trans. Inst. Chem. Eng. Part B Proc. Saf. Environ. Prot..

[15] Low K.S., Lee C.K., Lee K.P. (1993). Sorption of copper by dye-treated oil-palm fibers. Bioresour. Technol..

[16] King P., Srinivas P., Prasanna Kumar Y., Prasad V.S.R.K. (2006). Sorption of copper(II) ion from aqueous solution by *Tectona grandis L.f.* (teak leaves powder). J. Hazard. Mater..

[17] Dąbrowski A., Hubicki Z., Podkościelny P., Robens E. (2004). Selective removal of the heavy metal ions from waters and industrial wastewaters by ion-exchange method. Chemosphere.

[18] Pehlivan E., Altun T. (2007). Ion-exchange of Pb^2+^, Cu^2+^, Zn^2+^, Cd^2+^, and Ni^2+^ ions from aqueous solution by Lewatit CNP 80. J. Hazard. Mater..

[19] Pan B., Qiu H., Pan B., Nie G., Xiao L., Lv L., Zhang W., Zhang Q., Zheng S. (2010). Highly efficient removal of heavy metals by polymer-supported nanosized hydrated Fe(III) oxides: Behavior and XPS study. Water Res..

[20] Kołodyńska D., Krukowska-Bąk J., Kazmierczak-Razna J., Pietrzak R. (2017). Uptake of heavy metal ions from aqueous solutions by sorbents obtained from the spent ion exchange resins. Microporous Mesoporous Mater..

[21] Araucz K., Aurich A., Kołodyńska D. (2020). Novel multifunctional ion exchangers for metal ions removal in the presence of citric acid. Chemosphere.

[22] Kołodyńska D., Skwarek E., Hubicki Z., Janusz W. (2009). Effect of adsorption of Pb(II) and Cd(II) ions in the presence of EDTA on the characteristics of electrical double layers at the ion exchanger/NaCl electrolyte solution interface. J. Colloid Interface Sci..

[23] Kołodyńska D., Sofińska-Chmiel W., Mendyk E., Hubicki Z. (2014). Dowex M 4195 and Lewatit TP 220 in heavy metal ions removal from acidic streams. Sep. Sci. Technol..

[24] Sofińska-Chmiel W., Kołodyńska D. (2016). Application of ion exchangers for the purification of galvanic wastewater from heavy metals. Sep. Sci. Technol..

[25] Hubicki Z., Kołodyńska D., Ayben K. (2012). Selective removal of heavy metal ions from waters and waste waters using ion exchange methods. Ion Exchang. Technol.

[26] Shi J., Yi S., He H., Long C., Li A. (2013). Preparation of nanoscale zero-valent iron supported on chelating resin with nitrogen donor atoms for simultaneous reduction of Pb^2+^ and NO_3−_. Chem. Eng. J..

[27] Gao J., Liu F., Ling P., Lei J., Li L., Li C., Li A. (2013). High efficient removal of Cu(II) by a chelating resin from strong acidic solutions: Complex formation and DFT certification. Chem. Eng. J..

[28] Hamabe Y., Hirashima Y., Izumi J., Yamabe K., Jyo A. (2009). Properties of a bifunctional chelating resin containing aminomethylphosphonate and sulfonate derived from poly(x-bromobutylstyrene-co-divinylbenzene) beads. React. Funct. Polym..

[29] Dragan E.S., Dinu M.V., Lisa G., Trochimczuk A.W. (2009). Study on metal complexes of chelating resins bearing iminodiacetate group. Eur. Polym. J..

[30] Stevens J.S., Byard S.J., Seaton C.C., Sadiq G., Davey R.J., Schroeder S.L.M. (2014). Proton transfer and hydrogen bonding in the organic solid state: A combined XRD/XPS/ssNMR study of 17 organic acid–base complexes. Phys. Chem. Chem. Phys..

[31] Gobbo P., Novoa S., Biesinger M.C., Workentin M.S. (2013). Interfacial strain-promoted alkyne-azidecycloaddition (I-SPAAC) for the synthesis of nanomaterial hybrids. Chem. Commun..

[32] Gobbo P., Mossman Z., Nazemi A., Niaux A., Biesinger M.C., Gilles E.R., Workentin M.S. (2014). Versatile stained alkyne modified water-soluble aunps for interfacial strain promoted azide-alkyne cycloaddition (ISPAAC). J. Mater. Chem. B.

[33] Wanger C., Riggs W., Davis L., Moulder J., Muilenberg G. (1979). Handbook of X-ray Photoelectron Spectroscopy.

[34] https://www.lenntech.com/Data-sheets/Dowex-M-4195-L.pdf.

[35] Wagner C.D., Naumkin A.V., Kraut-Vass A., Allison J.W., Powell C.J., Rumble J.R. NIST Standard Reference Database 20, Version 3.4; NIST X-ray Photoelectron Spectroscopy Database; 2003. http:/srdata.nist.gov/xps/.

[36] Watts J., Wolstenholme J. (2003). An Introduction to Surface Analysis by XPS and AES.

[37] Gobbo P., Biesinger M.C., Workentin M.S. (2013). Facile synthesis of gold nanoparticle (AuNP)-carbonnanotubes (CNT). Hybrids through an interfacial Michael addition reaction. Chem. Commun..

[38] Beamson G., Briggs D. (1992). High Resolution XPS of Organic Polymers-the Scienta ESCA300 Database.

[39] Mohtasebi A., Chowdhury T., Hsu L.H.H., Biesinger M.C., Kruse P. (2016). Interfacial charge transfer between phenyl-capped aniline tetramer films and iron oxide surfaces. J. Phys. Chem. C.

